# The in vivo preventive and therapeutic properties of curcumin in bile reflux‐related oncogenesis of the hypopharynx

**DOI:** 10.1111/jcmm.15640

**Published:** 2020-07-21

**Authors:** Sotirios G. Doukas, Panagiotis G. Doukas, Clarence T. Sasaki, Dimitra Vageli

**Affiliations:** ^1^ The Yale Larynx Laboratory Department of Surgery Yale School of Medicine New Haven CT USA

**Keywords:** *Bcl2*, bile, curcumin, *Egfr*, hypopharyngeal cancer, laryngopharyngeal reflux, NF‐κB, *Stat3*

## Abstract

Bile at strongly acidic pH exerts a carcinogenic effect on the hypopharynx, based upon recent pre‐clinical studies that support its role as an independent risk factor. We recently demonstrated in vitro that curcumin can prevent oncogenic profile of bile in human hypopharyngeal cells, by inhibiting NF‐κB. We hypothesize that topically applied curcumin to the hypopharynx can similarly block early oncogenic molecular events of bile, by inhibiting NF‐*κ*B and consequently altering the expression of genes with oncogenic function. Using *Mus musculus* (C57Bl/6J), we topically applied curcumin (250 μmol/L; three times per day; 10 days) to the hypopharynx, 15 minutes before, 15 minutes after or in combination with bile acids (pH 3.0). Immunohistochemical analysis and qPCR revealed that topically applied curcumin either before, after or in combination with acidic bile exposure significantly suppressed its induced NF‐κB activation in regenerating epithelial cells, and overexpression of *Rela, Bcl2, Egfr, Stat3, Wnt5a, Tnf, Il6, Ptgs2. Akt1* was particularly inhibited by curcumin when applied simultaneously with bile. We provide novel evidence into the preventive and therapeutic properties of topically applied curcumin in acidic bile‐induced early oncogenic molecular events in hypopharyngeal mucosa, by inhibiting NF‐κB, and shaping future translational development of effective targeted therapies using topical non‐pharmacologic inhibitors of NF‐κB.

## 1 INTRODUCTION

Hypopharyngeal cancer represents one of the most aggressive subtypes of head and neck cancer. Although tobacco and alcohol have been associated with hypopharyngeal carcinogenesis, recent epidemiologic studies also support the conclusion that gastro‐oesophageal reflux disease (GERD) is an independent risk factor.^1^, ^2^ The prevalence of GERD has increased significantly in the United States over the last two decades, ranging from 18.1% to 27.8%. Moreover, biliary reflux a variant of GERD is not uncommon, leading to inflammatory and neoplastic events.^3^, ^4^, ^5^, ^6^ Recent in vitro studies showed that a mixture of conjugated bile acids, previously found in GERD patients,^5^, ^7^ in combination with acid, could promote a prolonged NF‐κB activation accompanied by a variety of molecular alterations, including overexpression of genes previously associated with head and neck cancer.^8^, ^9^ Using an in vivo animal model, it was also previously demonstrated that the same bile mixture could enhance the activation of NF‐κB, inducing pre‐neoplastic events and invasive hypopharyngeal squamous cell carcinoma, related to early oncogenic molecular changes (overexpression of genes like *Rela(p65), Bcl2, Tnf, Egfr, Stat3, Il6* and deregulation of miRNAs), in hypopharyngeal epithelium.^10^, ^11^, ^12^ Interestingly, recently published translational data have confirmed that hypopharyngeal cancer specimens derived from patients with bile‐containing gastro‐oesophageal refluxate, demonstrate an enhanced NF‐κB activation and expression of similar molecular profiles as those identified in our murine model.^13^

We recently demonstrated that the in vitro application of curcumin on human hypopharyngeal primary cells can block the NF‐κB activated oncogenic pathway induced by acidic bile,^14^ as has been similarly demonstrated using a pharmacologic inhibitor of NF‐κB (BAY 11‐7082).^15^, ^16^, ^17^ These data support curcumin as an alternative NF‐κB inhibitor that can be used to prevent bile reflux‐related early pre‐neoplastic events.

It has been shown that NF‐κB can be constitutively activated in squamous cell carcinomas, including head and neck, playing a key role in the neoplastic transformation of inflammatory epithelium.^18^, ^19^ It has also been shown that curcumin has anti‐cancer and chemo‐preventive properties,^20^, ^21^ by inhibiting NF‐κB and suppressing the growth of cancer cells.^22^ Although the precise mechanism by which curcumin blocks the NF‐κB anti‐apoptotic pathway is not completely understood, it has been documented that curcumin inhibits nuclear translocation of NF‐κB by blocking the phosphorylation and degradation of IκBα, and as a result prevents the transcriptional activation of the NF‐κB target genes involved in oncogenesis.^21^, ^23^, ^24^, ^25^

Curcumin therapeutic potential has been supported by previous studies.^22^, ^26^, ^27^, ^28^, ^29^, ^30^ The systematic administration of curcumin has some limitations due to its poor absorption, bio‐distribution, metabolism and bioavailability.^26^ For that reason, various formulations have made to increase the bioavailability of curcumin and improve curcumin's therapeutic applications, using nanoparticles, liposomes, cyclic oligosaccharides ^26^, ^27^, ^28^ or intraperitoneal injection.^29^, ^30^ On the other hand, it has previously been reported that topical application of curcumin to head and neck cancer in mice may effectively inhibit tumour growth.^22^

Here, we hypothesize that in vivo short‐term topically applied curcumin on murine hypopharyngeal mucosa (HM) before, after or in combination with acidic bile exposure is capable of suppressing the early pre‐neoplastic molecular events induced by acidic bile. To explore the temporal characteristics of curcumin inhibition, we used a previously established murine in vivo model of short‐term intermittent topical administration on HM.^17^ The effectiveness of topically applied curcumin in suppressing the acidic bile‐induced oncogenic effect could support future clinical trials, exploring the role of curcumin in the prevention and treatment of bile reflux‐related hypopharyngeal neoplasia.

## 2 MATERIALS AND METHODS

### 2.1 Animal model

Forty‐eight mice, *Mus musculus*, C57BL/6J (Jackson Laboratory, USA) (24‐male and 24‐female), eight per group (4‐male + 4‐female), were used in our experiment. Procedures followed the approved protocol 11039 by Institutional Animal Care and Use Committee (IACUC, Yale University). Mice were housed and husbanded by Yale Animal Resources Centre (YARC) having access to food and water throughout the day. According to the approved protocol, the animals were acclimatized 3 days before treatment procedures. The treatment procedures included topical exposure of HM, using a plastic feeding tube, to either experimental or control fluids, three times per day for ten days (with a 6‐hour interval in which the animals had access to drinking water, ensuring adequate washout between treatments).^10^, ^17^ We used 10 mmol/L of a mixture of bile salts (Sigma, St. Louis, MO, USA; Calbiochem, San Diego, CA, USA) at molar concentrations previously described,^10^ and considered to be close to those measured in gastro‐oesophageal refluxate of GERD patients.^5^, ^7^ The bile mixture was brought to pH 3.0 with 1 mol/L HCl (using a pH metre) before its application. We selected pH 3.0 that was previously found to induce a significant activation of NF‐κB and its associated oncogenic gene expression profile and to produce pre‐cancerous lesions in murine hypopharynx.^10^ We also used curcumin 250 μmol/L (~2 mg/kg/d; ≥94% curcuminoid content; ≥80% curcumin; Sigma‐Aldrich), previously found to be effective when topically applied using DMSO as a vehicle (DMSO final concentration ≤0.1%).^22^ Curcumin was topically applied to the hypopharynx three times per day for 10 days, (with a 6‐hour an interval), using a plastic feeding tube, as described below:

Treated groups included (a) BA (acidic bile): bile at pH 3.0, (b) Pre‐Cur: pre‐application of curcumin 15 minutes before acidic bile exposure, (c) Co‐Cur: co‐administration of acidic bile plus curcumin, (d) Post‐Cur: post‐application of curcumin 15 minutes after acidic bile exposure (e) Control: a treated control group using saline with DMSO at pH 7.0 [control for the effect of the feeding tube on the hypopharynx and the vehicle of curcumin (DMSO)] and (f) UC: an untreated group was included as negative control. We executed all the treatment procedures following the IACUC guidelines and policy (protocol 11039).

Towards the end of the 10th day, approximately 2 hours after the last treatment, the mice were killed using CO_2_ (IACUC guidelines) and kept on ice. The dissected HM of 4 animals/group (2 males + 2 females) was immediately placed into 10% neutral buffered formalin and submitted to Yale Pathology Facilities for histologic and immunohistochemical analysis after tissue embedding in paraffin blocks (FFPE). The HM of the remaining 4 animals of each group was placed in RNA stabilization solution (RNAlater^®^, Life Technologies, Carlsabad, CA, USA) and stored in − 80°C freezer, for RNA isolation.

### 2.2 Histologic evaluation

Light microscopy was performed in 3‐ to 4‐μm‐thick tissue sections of formalin‐fixed and paraffin‐embedded (FFPE) HM from experimental and control groups that were stained with haematoxylin and eosin. The tissue specimens were examined carefully to exclude haemorrhagic or ulcerative lesions that could indicate local toxicity caused by the use of the application method.

Although significant histologic changes were not expected in 10‐day acidic bile‐treated HM, histopathologic evaluation was also assessed as previously described.^10^, ^17^

### 2.3 Immunohistochemical (IHC) analysis

We microscopically examined the activated levels of NF‐*κ*B in exposed HM, using an IHC chromogenic staining for p‐NF‐*κ*B, as previously described,^10^, ^11^, ^12^, ^13^, ^17^ by documenting nuclear localization of phospho‐NF‐*κ*B in hypopharyngeal mucosal layers and its expression levels, under acidic bile exposure with or without topically applied curcumin. Specifically, IHC analysis was performed to determine whether the topical application of curcumin on HM was capable of reducing the effect of acidic bile (a) to induce NF‐κB activation, by preventing its nuclear localization in basal/parabasal and suprabasal layers, and (b) to promote regenerative activity, by suppressing Ki67 overexpression in basal/parabasal layers. We performed chromogenic IHC analysis, using immunoperoxidase (DAB peroxidase substrate), in serial sections (4 μm) of FFPE HM of 4 animals (2 males and 2 females) per group, to detect activation of NF‐κB and underlying molecular changes supporting cell proliferation in HM, as previously described.^10^, ^17^ A rabbit polyclonal anti‐phospho‐p65 Ser536 antibody was used to detect nuclear p‐p65 S536 localization, identifying activated NF‐κB (AbD Serotec, BIO‐RAD, Hercules, CA, USA), whereas a rabbit monoclonal anti‐Ki67 antibody was used to detect cell proliferation marker Ki67 (SP6, Thermo Scientific™ Lab Vision, Loughborough, UK). Leica light microscope and Aperio CS2 were used to examine the slides and capture images. Image Scope software was used for image analysis.

Two independent images per tissue section (at least four tissue sections per group) were analysed, and nuclear p‐p65 (S536) and Ki67 protein levels were expressed as ratios of positive nuclei to the total number of nuclei (defined as positivity), as we previously described.^17^ The expression levels (nuclear positivity) of the analysed markers were performed as follows: (a) acidic bile alone versus saline‐treated HM (b) curcumin versus acidic bile alone treated HM (mean ± SD by multiple *t* test). Untreated mucosa was used as a negative control.

### 2.4 Determination of gene expression profiles

Real‐time quantitative PCR analysis was performed to evaluate the effect of topically applied curcumin on HM, in inhibiting the acidic bile‐induced overexpression of genes previously associated with hypopharyngeal cancer‐related mRNA phenotype,^8^, ^12^ and that was found to be inhibited by the in vitro application of curcumin.^13^ The analysis included the mRNA expression of *Rela (p65), Egfr, Stat3, Il6, Tnf, Wnt5a* and *Bcl2*,^10^, ^17^ as well as of *Ptgs2, Mtor* and *Akt1*. Total RNA was isolated from HM (preserved in RNAlater and stored at −80°C) of 4 animals (2 males and 2 females) per group (RNeasy plus kit; Qiagen Inc, Valencia, CA, USA), and its quality and concentration were determined using a NanoDrop™ 1000 spectrophotometer [absorption ratios at 260/280 nm (>2.0) and 260 nm, respectively] (Thermo Fisher Scientific). After reverse transcription (iScript cDNA synthesis kit; Bio‐Rad), we performed real‐time qPCR analysis (CFX96™; Bio‐Rad) using murine specific primers for target and reference genes (Table S1) (QuantiTect Primers Assays and iQ™ SYBR Green Supermix by Bio‐Rad), as described previously.^10^, ^16^ The relative transcriptional levels were expressed as ratios of mRNA levels of each target gene relative to the reference gene (ΔΔCt). Data analysis was performed using CFX96™ software (Bio‐Rad) for qPCR analysis.

GraphPrism 7 software (GraphPad Software, San Diego, CA, USA) was used to perform one‐way ANOVA (Friedman or Kruskal‐Wallis; Dunn's multiple analysis test) and *t* test analysis (multiple comparisons by Holm‐Sidak) to assess the statistical significance of our data (*P* values < 0.05).

## 3 RESULTS

### 3.1 In vivo topically applied curcumin blocks the activation of NF‐κB induced by acidic bile

Immunohistochemical analysis revealed that pre‐treatment, simultaneous co‐administration or post‐treatment of curcumin successfully prevented or respectively suppressed, the activation of NF‐κB induced by acidic bile. This was demonstrated microscopically by the decreased nuclear detection of p‐NF‐*κ*B (p65 SS536) in hypopharyngeal mucosal compartments and the reduced nuclear positivity of p‐NF‐*κ*B (ratios of positive nuclei to the total number of nuclei).

**FIGURE 1:**
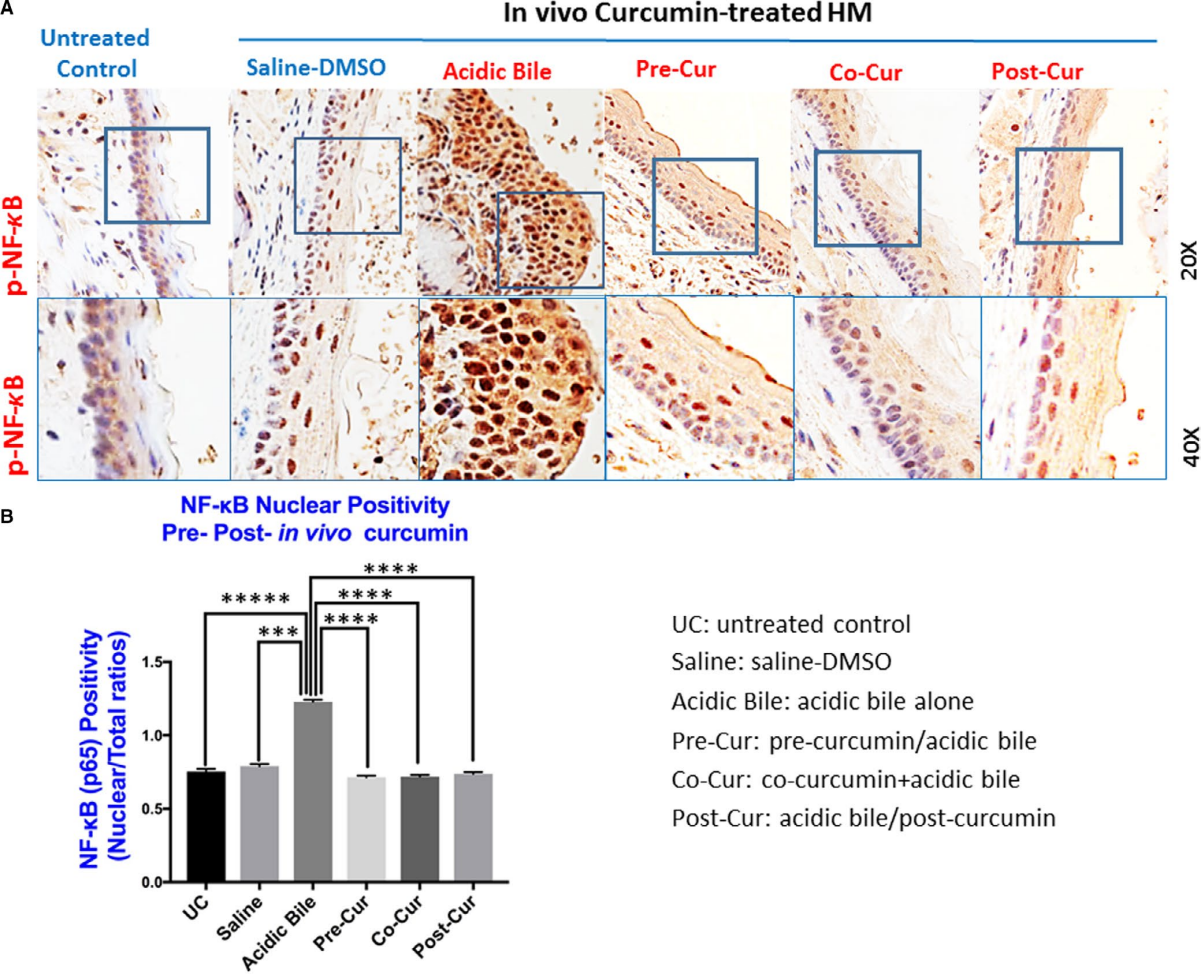
In vivo topical application of curcumin inhibits acidic bile‐induced activation of NF‐kB. A, IHC analysis for p‐NF‐κB (p65 S536) (from left to right): *control untreated HM*, showing cytoplasmic staining; *saline‐DMSO–*treated HM, incorporating sporadic and weak cytoplasmic or nuclear staining in a few basal cells; acidic bile‐treated HM, inducing intense nuclear and cytoplasmic staining of basal and parabasal/suprabasal layers; *Pre‐Cur: pre‐application of curcumin + acidic bile‐treated HM*, showing weak nuclear or cytoplasmic staining of cells of basal or suprabasal layers; *Co‐Cur: co‐application of curcumin + acidic bile‐treated HM*, demonstrating cytoplasmic staining of cells of basal layer and weak nuclear or cytoplasmic staining of sporadic cells of suprabasal layers; and *Post‐Cur: acidic bile‐treated HM + post‐application of curcumin*, demonstrating nuclear or cytoplasmic staining mainly of cells of basal layer and a weak nuclear or cytoplasmic staining of few cells in suprabasal layers. Images were captured using Aperio CS2 and analysed by Image Scope software (Leica Microsystems, Buffalo Grove, IL, USA). B, Nuclear positivity of p‐NF‐κB (p65 S536) in murine HM (*P* values by *t* test; multiple comparisons by Holm‐Sidak; GraphPad Prism 7.0; GraphPad Software, San Diego, CA, USA) (positivity = nuclear‐positive p‐p65 staining/total number of nuclei). Acidic bile (pH 3.0) induces significantly higher positive nuclear p‐NF‐κB (p65 S536) levels compared to saline‐DMSO–treated HM or untreated control. Topical pre‐, post‐ or co‐application of curcumin significantly decreases nuclear p‐NF‐κB levels in acidic bile (pH 3.0) HM (*P* values by *t* test; multiple comparisons by Holm‐Sidak; GraphPad Prism 6.0)

Specifically, we observed that HM treated with curcumin produced weak nuclear staining of p‐NF‐κB (p65 SS536) in basal/suprabasal layers (Figure 1A). On the other hand, similar to prior findings,^16^ we observed intense nuclear staining of p‐NF‐κB (p65 S536) in basal/parabasal and suprabasal layers in HM exposed to acidic bile alone compared to controls (Figure 1A), indicating an enhanced activation of NF‐κB.

Scoring of nuclear p‐NF‐κB (p65 S536) revealed that HM treated with curcumin, before, after or in combination with acidic bile, demonstrated significantly lower levels of activated NF‐κB compared to HM treated with acidic bile alone (Figure 1B) (*P* < 0.05, by *t* test). However, we found significantly higher nuclear levels of p‐NF‐κB in HM treated with acidic bile alone compared to controls, similar to our prior findings (Figure 1B).^17^

Saline‐DMSO–treated HM that was used as a reference control for mechanical stress of application showed a weak nuclear staining for p‐NF‐κB (Figure 1), indicating less NF‐κB activation, whereas the untreated HM was found negative for NF‐κB activation (Figure 1).

In general, microscopic examination, as well as scoring of p‐NF‐κB positivity, revealed that curcumin, whether it was applied before, after or in combination with acidic bile, caused a similar inhibitory effect on NF‐κB activation.

No significant difference was observed in p‐NF‐κB positivity and response to treatment between males and females.

### 3.2 In vivo topically applied curcumin prevents the increased Ki67 levels induced by acidic bile

**FIGURE 2:**
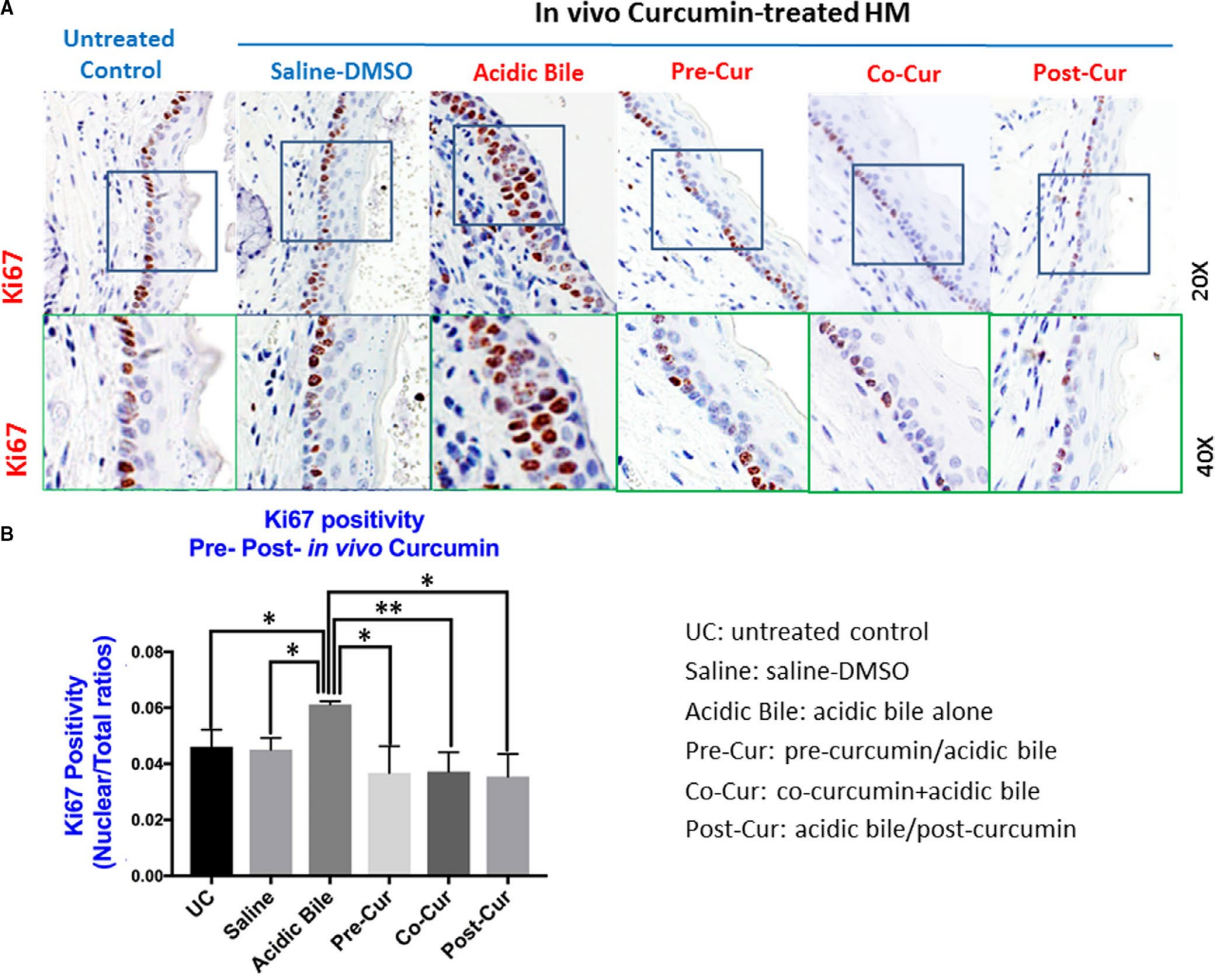
In vivo topical application of curcumin prevents acidic bile‐induced increased cell proliferation in treated HM. A, IHC analysis for Ki67 (from left to right): *control untreated HM*, showing weak cytoplasmic or nuclear staining in few basal cells; *saline‐DMSO–treated HM*, demonstrating sporadic and weak cytoplasmic or nuclear staining in cells of the basal layer; *acidic bile‐treated HM*, showing intense nuclear and cytoplasmic staining of cells of basal and parabasal/suprabasal layers; and *Pre‐Cur: pre‐application of curcumin + acidic bile‐treated HM, Co‐Cur: co‐application of curcumin + acidic bile‐treated HM*, and *Post‐Cur: acidic bile‐treated HM + post‐application of curcumin*, revealing weak nuclear or cytoplasmic staining in cells of basal layer. Images were captured using Aperio CS2 and analysed by Image Scope software (Leica Microsystems). B, Graphs depict acidic bile (pH 3.0) induces significantly higher positive nuclear Ki67 levels compared to saline‐DMSO–treated HM or untreated control. Topical pre‐, post‐ or co‐application of curcumin significantly decreases nuclear Ki67 levels in acidic bile (pH 3.0) HM (*P* values by *t* test; multiple comparisons by Holm‐Sidak; GraphPad Prism 7.0) (positivity = nuclear‐positive Ki67 staining/total number of nuclei)

Microscopic examination of short‐term (10‐day) acidic bile‐treated HM revealed intense staining of Ki67 in basal and parabasal layers compared to controls, consistent with increased cell proliferation (Figure 2A,B), similar to prior observations.^17^ In contrast, topical application of curcumin before, after or in combination with acidic bile induced weak Ki67 staining in sporadic cells limited to the basal layer, similar to controls (saline‐DMSO–treated HM and untreated HM), indicating that curcumin successfully suppressed the regeneration of epithelial cells promoted by acidic bile exposure (Figure 2A).

Scoring of nuclear Ki67 revealed that HM exposed to curcumin developed significantly lower levels of nuclear Ki67 compared to HM treated with acidic bile alone (Figure 2B) (*P* < 0.05, by *t* test). On the other hand, we found that the repetitive short‐term topical application of acidic bile alone promoted increased nuclear Ki67 levels with a statistically significant difference compared to controls, an observation similar to prior studies (Figure 2B).^17^

No histologic signs of toxicity were found.^10^, ^17^

### 3.3 In vivo topical application of curcumin effectively inhibits the overexpression of the NF‐κB‐related genes with oncogenic function induced by acidic bile exposure

**FIGURE 3:**
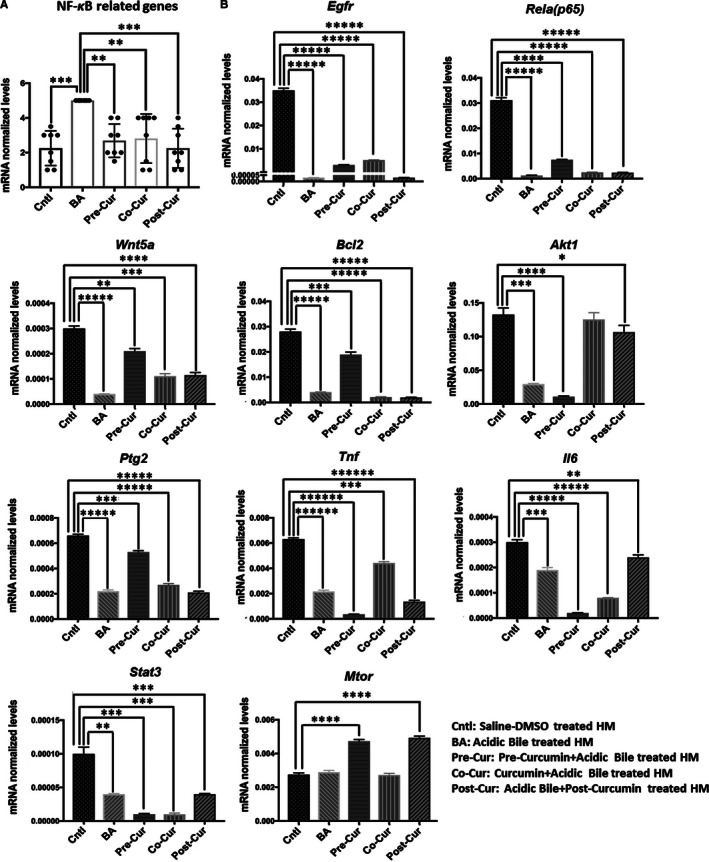
In vivo topical application of curcumin effectively inhibits the Acidic Bile‐induced overexpression of the NF‐κB‐Related genes with oncogenic function. A, Transcriptional levels of the analysed NF‐κB‐related genes with oncogenic function are depicted in murine HM topically exposed to control saline‐DMSO, acidic bile alone and to curcumin 15 min before (Pre‐Cur), simultaneously (Co‐Cur) or 15 min after (Post‐Cur) acidic bile exposure. Graphs created by GraphPad Prism 7 software (transcriptional levels of the analysed genes are normalized to *Gapdh* by real‐time qPCR analysis). (One‐way ANOVA, Freidman test). B, Graphs, created by Graph Pad Prism 7.0 software, show transcriptional levels (normalized to *Gapdh*) for each analysed gene, comparing HM exposed to control saline‐DMSO, acidic bile alone, and application of curcumin before, simultaneously or after acidic bile by real‐time qPCR analysis (*P* value < 0.05; by *t* test; multiple comparisons by Holm‐Sidak; data obtained from four analysed samples)

Quantitative PCR analysis revealed that topically applied curcumin before, after or in combination with acidic bile exposure successfully inhibited the overexpression of the NF‐κB‐related genes with anti‐apoptotic or oncogenic function induced by the acidic bile exposure (Figure 3A). Previous studies documented that acidic bile could induce significant overexpression of the analysed genes in short‐term (7‐days) treated HM,^17^ as well as in long‐term exposed pre‐malignant HM.^10^, ^12^ Here, we have documented that acidic bile‐induced overexpression of *Egfr, Rela, Wnt5a, Bcl2, Ptgs2, Tnf, Stat3* and *Il6* was effectively inhibited by topically applied curcumin (3, 4). Specifically, we showed that HM exposed to curcumin before, after or in combination with acidic bile, produced significantly lower mRNA levels of the analysed genes compared to HM exposed to acidic bile alone (Figure 3B) (*P* < 0.05; multiple *t* test).

**FIGURE 4:**
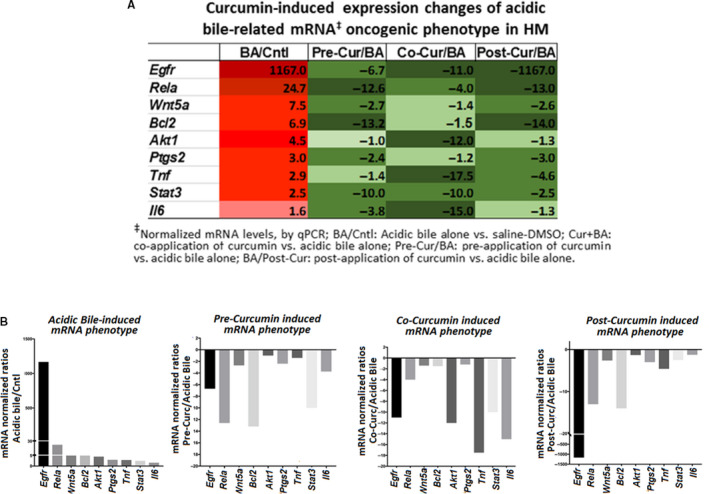
In vivo topical application of curcumin induces changes in the mRNA oncogenic phenotype, caused by acidic bile in a short‐term exposed murine HM. A, Table demonstrates the changes of mRNA oncogenic phenotype (fold changes of mRNAs), in short‐term exposed HM to BA/Cntl (acidic bile alone vs. control saline‐DMSO), Pre‐Cur/BA (pre‐application of curcumin + acidic bile vs acidic bile alone), Co‐Cur/BA (co‐application of curcumin + acidic bile vs acidic bile alone) and Post‐Cur/BA (acidic bile + post‐application of curcumin vs acidic bile alone). B, Columns of graphs created by GraphPad Prism software 7.0 depict mRNA phenotype in short‐term exposed HM to (A) acidic bile alone vs. control saline‐DMSO–treated HM), (B) pre‐application of curcumin vs acidic bile alone, (C) co‐application of curcumin vs acidic bile alone and (D) post‐application of curcumin vs acidic bile alone. mRNA phenotypes correspond to normalized transcriptional expression ratios of NF‐*κ*B‐related genes *Egfr, Rela (p65), Wnt5a, Bcl2, Akt1, Ptgs2*, *Tnf, Stat3* and *Il6*, by real‐time qPCR analysis. Pre‐, co‐ or post‐application of curcumin reverses the acidic bile‐induced mRNA phenotype

It is worthy to note that either pre‐ or post‐application of curcumin demonstrated a similar inhibitory effect on *Rela, Wnt5a, Bcl2* and *Ptgs2* that was found significantly more pronounced than occurred with its simultaneous co‐administration (3, 4). On the other hand, we found that post‐application of curcumin could induce a particularly stronger effect in suppressing acidic bile‐induced *Egfr* transcriptional activation, which was found to be significantly more intense compared to its pre‐ or co‐application (3, 4).

Although short‐term contact of acidic bile with HM produced a significant transcriptional activation of NF‐κB‐related genes, similar to prior observation, acidic bile exposure for this period was not capable of up‐regulating *Mtor* but could induce significant overexpression of *Akt1*, known factors in head and neck cancer (Figure 3B; Table S2). Simultaneous co‐application of curcumin with acidic bile significantly reversed *Akt1* overexpression, whereas pre‐application had a minimal effect and its post‐application had some inhibitory effect although significantly less than its simultaneous co‐administration with acidic bile (3, 4). Both pre‐application and post‐application accelerated the *Mtor* mRNAs compared to acidic bile alone (Figure 3B).

Spearman correlation revealed a significant linear correlation between NF‐κB inhibition‐induced mRNA levels of *Rela* and *Egfr (r* = 0.872,* P* = 0.05),* Rela* and *Wnt5a* (*r* = 0.9,* P* = 0.0417),* Rela* and *Ptgs2* (*r* = 0.9,* P* = 0.0417),* Stat3 and Il6* (*r* = 0.9487,* P* = 0.033),* Egfr* and *Ptgs2* (*r* = 0.8721,* P* = 0.049), and* Tnf and Akt1* (*r* = 0.9,* P* = 0.0417) (one tail). The identified strong linear correlations could imply possible interactions among the analysed genes under the effect of curcumin in acidic bile‐exposed murine HM, as previously suggested by the use of BAY 11‐7082.^17^

**FIGURE 5:**
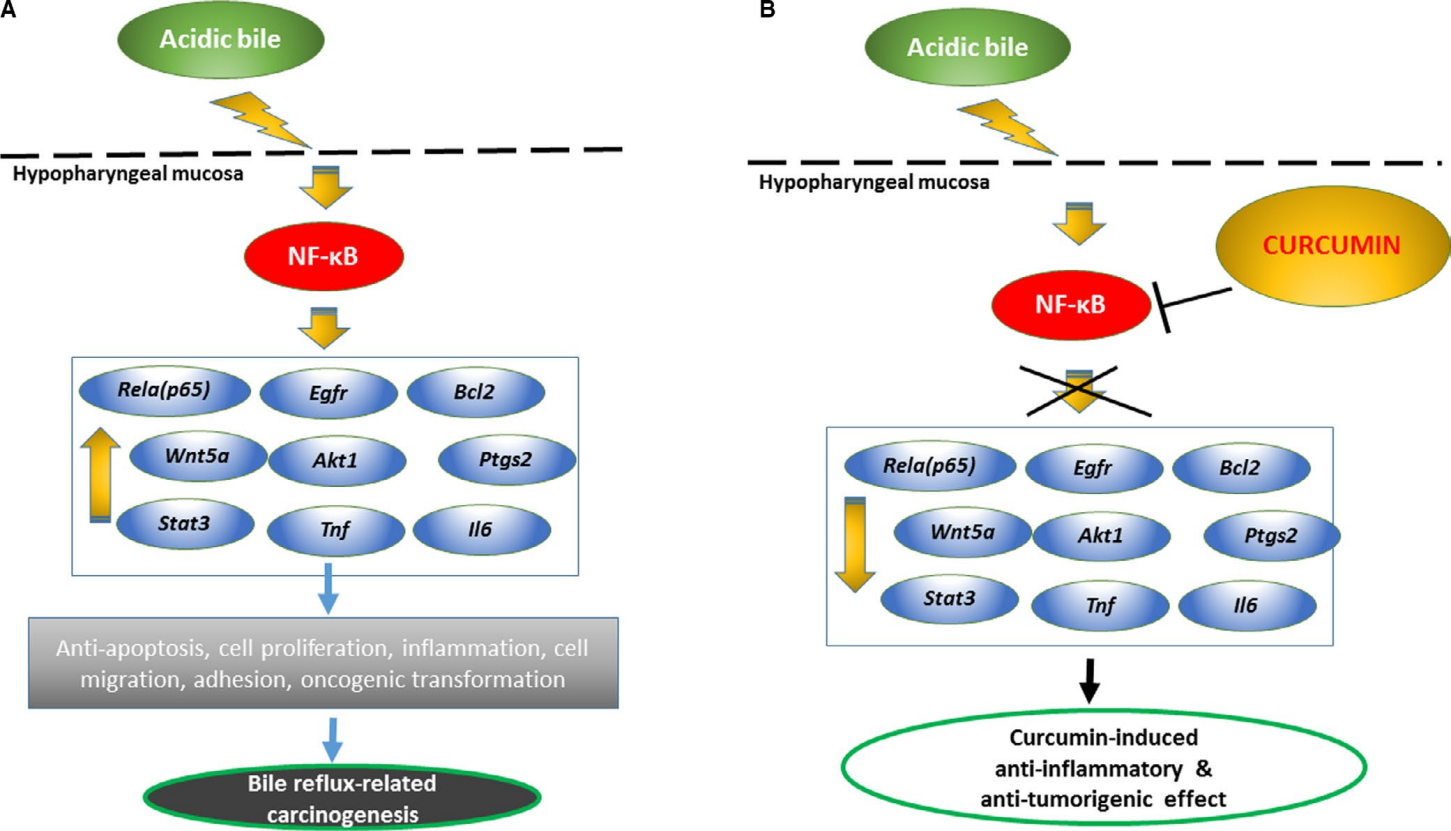
Schematic representation of in vivo prevention and therapeutic properties of curcumin on acidic bile‐induced hypopharyngeal carcinogenesis. A, Schematic representation of acidic bile‐induced NF‐κB activation and production of cancer‐related mRNA phenotype in short‐term (10‐d) exposed murine HM. B, Schematic representation of inhibition of acidic bile‐induced activation of NF‐κB and its related genes with anti‐apoptotic or oncogenic function by topical application of curcumin on murine HM, before, simultaneously or after acidic bile exposure

In general, qPCR analysis revealed a reversed mRNA phenotype in HM treated with curcumin before, after or in combination with acidic bile, compared to HM treated with acidic bile alone (Figure 4B), supporting the observation that topically applied curcumin significantly inhibited the acidic bile‐induced transcriptional activation of genes known to play a central role in head and neck malignancies (Figure 5),^8^, ^9^, ^10^, ^12^ similar to the effect induced by topically applied pharmacologic NF‐κB inhibitor (BAY 11‐7082).^17^

No significant difference was found in gene expression and response to treatment between male and female mice.

## 4 DISCUSSION

During reflux events, gastric (acid) and duodenal (bile) contents are capable of contacting supra‐oesophageal mucosa with potential carcinogenic effect.^1^ Although anti‐acid therapy remains the first‐line treatment for the prevention of reflux‐induced inflammation, novel data openly question its effectiveness in preventing reflux‐related malignancies long term,^31^ paving the way for alternative or additional evidence‐based preventive approaches. Because extra‐oesophageal reflux events are clinically random in distribution, the application of a therapy synchronized to each reflux episode may not clinically practical.^32^ Therefore, demonstrating that effects of a topical treatment either before or after the reflux event would be largely equivalent to its simultaneous co‐administration, could plausibly support a clinically effective regimen of topical pharmacologic management.

Recent studies demonstrated that repetitive exposure of hypopharyngeal mucosa to bile at acidic pH could induce activation of NF‐*κ*B and deregulation of oncogenic factors and subsequent pre‐malignant and malignant changes.^10^, ^11^, ^12^ Specifically, we previously showed that acidic bile‐induced pre‐malignant and malignant lesions of murine HM demonstrated intense nuclear staining of p‐NF‐*κ*B, using IHC analysis, a well established morphologic method to document activation of NF‐*κ*B,^33^ accompanied by a significant overexpression of NF‐*κ*B‐related mRNA oncogenic phenotype, compared to controls.^10^, ^12^, ^13^ On the other hand, the topical co‐administration of specific pharmacologic NF‐κB inhibitor BAY 11‐7082 was capable of preventing early pre‐neoplastic molecular changes induced by acidic bile exposure.^17^ In addition, it has recently been demonstrated that in vitro concurrent application of a dietary NF‐κB inhibitor such as curcumin with acidic bile sufficiently blocked the acidic bile‐induced tumorigenic effect on hypopharyngeal primary cells, by eliminating NF‐κB activation and preventing the transcriptional activation of molecules central to head and neck cancer.^14^, ^19^, ^34^, ^35^, ^36^

Although curcumin has a limited systemic effect due to its poor absorption, it has been shown to be effective when applied topically.^21^, ^22^, ^37^, ^38^ To our knowledge, this is the first exploration of topically applied curcumin on acidic bile‐exposed HM to demonstrate its preventive and therapeutic properties on acidic bile‐induced early oncogenic molecular events. Using a similar methodology to those used in our prior animal and human studies,^10^, ^11^, ^12^, ^13^, ^17^ we demonstrated the effect of curcumin to significantly reduce the acidic bile‐induced NF‐*κ*B activation in HM. The essential role of NF‐*κ*B in cancer has been previously established by mediating the transcriptional activation of target genes, known to be involved in oncogenic pathways, such as TNF‐α/NF‐κB, EGFR/Ras/RAF/MAPK, Akt/PI3K/mTOR, ΙΚΚ/NF‐κB, STAT3 and wnt/β‐catenin,^19^, ^39^, ^40^, ^41^ and linked to head and neck cancer.^39^, ^40^ Furthermore, we previously showed that hypopharyngeal squamous cell carcinoma obtained from patients with documented biliary laryngopharyngeal reflux demonstrated significant transcriptional activation of NF‐*κ*B‐related oncogenic factors,^13^ similarly found in our pre‐clinical in vitro and in vivo models and negatively affected by a pharmacologic inhibitor of NF‐*κ*B.^8^, ^9^, ^10^, ^11^, ^14^, ^15^, ^16^, ^17^ In this study, we, therefore, seek to address the topical effect of curcumin in blocking the previously characterized bile‐related mRNA oncogenic phenotype of NF‐*κ*B target genes, in murine HM. The novel findings of this in vivo model are in line with previous data from an in vitro model, exploring the effect of curcumin in human hypopharyngeal primary cells, in inhibiting the acidic bile‐induced activation of NF‐*κ*B and its related transcriptional activity,^14^ and give insights into the prevention and development of effective targeted therapies using non‐pharmacologic NF‐κB inhibitors, in bile reflux‐related early pre‐neoplastic molecular events. Specifically, our current findings demonstrate that topically applied curcumin on HM, either before, after or in combination with acidic bile, can effectively (a) inhibit the NF‐κB activation induced by the acidic bile exposure, throughout its mucosal thickness by IHC (Figure 1), (b) suppress cell proliferation in HM, as indicated by the decreased levels of Ki67 marker in its regenerating epithelial cells (Figure 2) and (c) block the activation of NF‐κB transcriptional factor *Rela(p65)* and oncogenic *Egfr, Stat3*, *Wnt5a*, *Bcl2*, *Tnf*, *Il6* and *Ptgs2* induced by acidic bile (Figure 3B). Our observations demonstrate that the pro‐oncogenic and anti‐oncogenic effect of acidic bile on HM can be prevented and suppressed by topically applied curcumin (Figure 5), in a way similarly observed by pharmacologic inhibitors,^17^ based on its previously established anti‐oncogenic and anti‐inflammatory properties.^21^, ^22^, ^23^, ^24^, ^25^

The observation that pre‐ or simultaneous co‐application of curcumin with acidic bile exerts a more significant effect on inhibition of *Stat3*, a head and neck cancer‐related transcriptional factor,^29^, ^37^, ^42^ than its post‐application, further supports the view that curcumin successfully blocks early cancer‐related effects of acidic bile on HM. It is also worth highlighting that either pre‐ co‐ or post‐application of curcumin effectively inhibits the acidic bile‐induced overexpression of *Egfr*, a central oncogenic factor in head and neck malignancies,^19^, ^34^ with a significantly more profound effect by its post‐application. This observation is supported by the apparent direct negative effect of curcumin on *Egfr* activation^43^, ^44^ that may be partially affected by its bioavailability,^26^ during the 15‐minute interval, when HM is exposed to acidic bile. Our data also show that the acidic bile‐induced *Akt1* overexpression, a frequent event in human cancers including head and neck,^45^ can be successfully inhibited by post‐treatment or simultaneous co‐administration of curcumin with acidic bile compared to its pre‐treatment. This observation supports the view that curcumin, by blocking constitutive NF‐κB activation may have a further suppressive effect on *Akt1* overexpression, in line with prior reports.^46^

Although timing of curcumin administration may affect the relative intensity of its preventive or therapeutic effect, likely reflecting the variable biological activities of component metabolites (eg tetrahydrocurcumin, hexahydrocurcumin and octahydrocurcumin),^26^ it is clear that topical administration of curcumin even if it were not to be precisely synchronized with each reflux event is overall capable of suppressing its induced oncogenic mRNA phenotype.

In summary, our in vivo model supports the conclusion that short‐term topical application of curcumin when administered either before, after or concurrently with acidic bile can effectively inhibit the activation of NF‐*κ*B and early pre‐neoplastic events on HM, induced by acidic bile exposure. Based on our recent findings, acidic bile has the potential for actively influencing the progression of hypopharyngeal cancer, mediated by NF‐*κ*B,^47^ as similar recently identified in hypopharyngeal squamous cell carcinomas from patients with biliary laryngopharyngeal reflux.^17^ We believe that apart from antacid therapy, the topical application of dietary inhibitors of NF‐*κ*B, like curcumin, is a crucial area for future research presenting a wide range of opportunities for potential therapeutic intervention in furthering control of bile‐related hypopharyngeal cancer recurrence and progression.

## 5 CONCLUSION

Our in vivo model confirms the observation that curcumin, when it is administered topically, can effectively block the bile reflux‐related early oncogenic molecular events in the hypopharynx, by inhibiting NF‐*κ*B, providing a basis for future translational inquiry.

## CONFLICT OF INTEREST

The authors confirm that there are no conflicts of interest.

## AUTHOR CONTRIBUTION

**Sotirios G. Doukas:** Conceptualization (lead); Data curation (equal); Formal analysis (equal); Investigation (lead); Methodology (lead); Supervision (lead); Validation (equal); Visualization (equal); Writing‐original draft (lead); Writing‐review & editing (lead). **Panagiotis G. Doukas:** Conceptualization (equal); Data curation (equal); Formal analysis (lead); Investigation (equal); Methodology (equal); Software (equal); Validation (equal); Visualization (equal); Writing‐original draft (equal); Writing‐review & editing (equal). **Clarence T. Sasaki:** Conceptualization (equal); Data curation (equal); Formal analysis (equal); Funding acquisition (lead); Investigation (equal); Project administration (lead); Resources (equal); Supervision (equal); Validation (equal); Writing‐original draft (equal); Writing‐review & editing (lead). **Dimitra P Vageli:** Conceptualization (lead); Data curation (lead); Formal analysis (equal); Investigation (lead); Methodology (lead); Resources (equal); Software (lead); Supervision (lead); Validation (equal); Visualization (equal); Writing‐original draft (lead); Writing‐review & editing (lead).

## ETHICAL APPROVAL

Methods were carried out in accordance with guidelines and regulations of Yale University. The in vivo experimental protocol was approved by Yale Institutional Animal Care & Use Committee (IACUC) (https://your.yale.edu/research‐support/animal‐research) (approved protocol 11 039 by IACUC of Yale University).

## Supporting information

Table S1‐S2

## Data Availability

The data support the findings of this study are available in the supplementary material of this article.
