# Supplementary material for: The in vivo preventive and therapeutic properties of curcumin in bile reflux‐related oncogenesis of the hypopharynx
Source: J Cell Mol Med. 2020 Jul 21;24(18):10311–21. doi: 10.1111/jcmm.15640 (PMC7521262; doi:10.1111/jcmm.15640)
Supplement: Supplementary file 1 — Table S1‐S2 [file JCMM-24-10311-s001.docx]

**Title: The in vivo preventive and therapeutic properties of curcumin in bile reflux-related oncogenesis of the hypopharynx.**

Sotirios G. Doukas^1^, Panagiotis G. Doukas^1^, Clarence T. Sasaki,^1^ Dimitra Vageli^1*^

^1^The Yale Larynx laboratory, Department of Surgery, Yale School of Medicine, New Haven, CT, USA

*corresponding author: **Dimitra P. Vageli**; The Yale Larynx laboratory, Department of Surgery (Otolaryngology), Yale School of Medicine, 310 Cedar Street (BML212), New Haven, CT 06510, USA; E-mail: [dimitra.vangeli@yale.edu](mailto:dimitra.vangeli@yale.edu); Tel: (203) 737 1447

**Supplementary Material**

**Supplementary Table S1:** Mouse genes (targets and *GAPDH*) and their detected transcripts, analyzed by real time qPCR, in murine HM.

| **Gene (mouse)** | **Detected transcripts** | **Amplicon length (bp)** |
| --- | --- | --- |
| *Gapdh* | NM_008084 | 144 |
|  | NM_001289726 |  |
| *Rela* | NM_009045 | 82 |
| *Stat3* | NM_011486 | 99 |
|  | NM_213659 |  |
|  | NM_213660 |  |
| *Wnt5a* | NM_001256224 | 130 |
|  | NM_009524 |  |
| *Bcl2* | NM_009741 | 80 |
| *Tnf* | NM_013693 | 112 |
|  | NM_001278601 |  |
| *Egfr* | NM_007912 | 68 |
|  | NM_207655 |  |
| *Il6* | NM_031168 | 128 |
| *Ptgs2* | NM_011198 | 95 |
| *Akt1* | NM_001165894, NM_009652, XM_006515417, XM_006515415, XM_006515416 | 69 |
| *Mtor* | NM_020009, XM_622902, XM_006539077 | 99 |

| **Target gene/**  ***Gapdh** (*ΔΔ^CT^*)** | ****Control** | **Acidic Bile** | **#Pre-Cur** | **‡Co-Cur** | **¥Post-Cur** |
| --- | --- | --- | --- | --- | --- |
| ***Egfr*** | 3.00E-05 | 3.50E-02 | 5.20E-03 | 3.11E-03 | 3.00E-05 |
| ***Rela*** | 1.26E-03 | 3.11E-02 | 2.47E-03 | 7.53E-03 | 2.37E-03 |
| ***Wnt5a*** | 4.00E-05 | 3.00E-04 | 1.11E-04 | 2.10E-04 | 1.15E-04 |
| ***Bcl2*** | 4.08E-03 | 2.80E-02 | 2.12E-03 | 1.90E-02 | 1.97E-03 |
| ***Akt1*** | 2.96E-02 | 1.33E-01 | 1.15E-01 | 1.10E-02 | 1.07E-01 |
| ***Ptgs2*** | 2.20E-04 | 6.60E-04 | 2.70E-04 | 5.30E-04 | 2.10E-04 |
| ***Tnf*** | 2.17E-03 | 6.30E-03 | 4.42E-03 | 3.60E-04 | 1.36E-03 |
| ***Stat3*** | 4.00E-05 | 1.00E-04 | 1.00E-05 | 1.00E-05 | 4.00E-05 |
| ***Il6*** | 1.90E-04 | 3.00E-04 | 8.00E-05 | 2.00E-05 | 2.40E-04 |
| ***Mtor*** | 2.90E-03 | 2.75E-03 | 2.72E-03 | 4.73E-03 | 4.93E-03 |

**Supplementary Table S2.**  Transcriptional levels of NF-κB related genes with oncogenic function in murine hypopharyngeal mucosa (HM).

* normalization of mRNA levels using *Gapdh*; **Saline-DMSO treated HM; # Pre-curcumin+Acidic bile treated HM; ‡Curcumin+Acidic Bile treated HM; ¥Acidic Bile+Post-Curcumin treated HM
